# The quality of outcome in anti‐reflux surgery is multi‐factorial

**DOI:** 10.1002/ueg2.12332

**Published:** 2022-11-03

**Authors:** Stephen E. Attwood

**Affiliations:** ^1^ Durham University Durham UK

The paper by Markar et al^1^ is an advisory consensus on the type of fundoplication that might be used in anti‐reflux surgery. Anti‐reflux surgery has taken criticism in the past decade due to a perception of poor results. This relate to concerns about side effects, dysphagia being the most frequently quoted, and the need for re‐operation. However, the certainty of evidence (see table 3)^1^ is “very low” and therefore application of this advice should be taken cautiously.

The need for a non‐drug therapy for treating reflux symptoms continues to raise new surgical and endoscopic options. It has long been accepted that the best results in anti‐reflux surgery come from centres that have frequent practice. High throughput of reflux patients improves case selection, fine tunes physiology departments in case selection,^2^ and trains the whole team ‐ surgeons, anaesthetists and nurses, to achieve a high standard. This is probably more important than the type of fundoplication. It has long been held that the surgeon is best doing the type of operation that he/she has been trained in, ensuring details of technique to be well practiced. For that reason in the Lotus trial, some years ago, when comparing outcomes of anti‐reflux surgery with long term dose adjusted proton pump inhibitor therapy, we chose surgeons with >20 anti‐reflux operations per annum and more than 40 such procedures in their individual experience.^3^ The outcomes of that study, using the Nissen posterior total fundoplication, showed a very low rate of complications, and of dysphagia (<5%), with a very low rate of recurrent hernia or re‐operation (<5%).^4^

The context of the study published in the United European Gastroenterology (UEG) in today's environment is influenced by an overall reduction in operative training of surgeons in their formative years. Many newly qualified surgeons have not had the hands‐on operative experience of previous generations. Choosing where to start in the process of developing anti‐reflux surgical technique becomes very relevant. The study of Markar informs us, based on published studies (not all randomised clinical trials, and rarely directly comparing one technique with another) that the “suggested technique of fundoplication should be a partial wrap and not a complete 360° wrap.

They are correct to make their statements as suggestions and not a standard of care because the evidence is not sufficiently strong. The advice clearly has value when commencing a career, but the authors also suggest that surgeons can adapt from a practice of using a 360° wrap. For this latter opinion there is no evidence, if the surgeon is already operating a high level of surgical quality. In my opinion it would be unwise to use this recommendation to criticise a surgeon with established practice, particularly if there is an audit of high quality outcome.

The difficulties encountered after anti reflux surgery are multi factorial and related to poor patient selection, inadequate explanation of outcomes, techniques of hiatal hernia repair that are prone to recurrence, wrap suturing techniques that predispose to complications and other factors. For instance, the use of fine threads to repair the crura (3/0 or finer) are associated with sutures cheese wiring through muscle, exacerbated if the surgeon pulls the knots tightly. A loosely sutured wide bore thread is less likely to result in a recurrence of hiatus hernia because the tissue is not strangulated and can grow together normally, circulated by working blood vessels. This applies to the sustainability of hiatus hernia repair as well as to the disruption of the wrap itself. Recurrence of reflux after anti reflux surgery has more to do with these factors than it has to do with the choice of fundoplication type. In the literature there is a high degree of variability in the reported rates of re‐do anti reflux surgery, and those rates do not associate with any particular type of fundoplication.

The UEG and European Association of Ensoscopic Surgery disclaimers that are included after the bibliography highlight the importance of the contextual interpretation of the study recommendations. They should not be used to blame a single bad outcome on a surgeon's chosen approach. This guidance should encourage future studies on outcomes to be carefully designed, and data prospectively collected. Modern surgery, and indeed all medical interventions should be the subject of prospective data collection, audit and standards improvement. The use of audit in a constructive non‐blame approach is likely to help to improve outcomes, and in the case of anti‐reflux surgery should be embarked on with guidance from professional bodies.

## Data Availability

No data is given in this Editorial.
